# Novel Inflammation-Based Prognostic Score for Predicting Survival in Patients with Metastatic Urothelial Carcinoma

**DOI:** 10.1371/journal.pone.0169657

**Published:** 2017-01-11

**Authors:** Yu-Li Su, Meng-Che Hsieh, Po-Hui Chiang, Ming-Tse Sung, Jui Lan, Hao-Lun Luo, Chun-Chieh Huang, Cheng-Hua Huang, Yeh Tang, Kun-Ming Rau

**Affiliations:** 1 Division of Hematology Oncology, Department of Internal Medicine, Kaohsiung Chang Gung Memorial Hospital and Chang Gung University, College of Medicine, Kaohsiung, Taiwan; 2 Clinical Trial Center, Kaohsiung Chang Gung Memorial Hospital, Kaohsiung, Taiwan; 3 Department of Urology, Kaohsiung Chang Gung Memorial Hospital and Chang Gung University, College of Medicine, Kaohsiung, Taiwan; 4 Department of Pathology, Kaohsiung Chang Gung Memorial Hospital and Chang Gung University, College of Medicine, Kaohsiung, Taiwan; 5 Department of Radiation Oncology, Kaohsiung Chang Gung Memorial Hospital and Chang Gung University, College of Medicine, Kaohsiung, Taiwan; 6 Division of Hematology Oncology, Department of Internal Medicine, E-Da Hospital, I-Shou University, Kaohsiung, Taiwan; Shanghai Jiao Tong University School of Medicine, CHINA

## Abstract

**Purpose:**

We developed a novel inflammation-based model (NPS), which consisted of a neutrophil to lymphocyte ratio (NLR) and platelet count (PC), for assessing the prognostic role in patients with metastatic urothelial carcinoma (UC).

**Materials and Methods:**

We performed a retrospective analysis of patients with metastatic UC who underwent systemic chemotherapy between January 1997 and December 2014 in Kaohsiung Chang Gung Memorial Hospital. The defined cutoff values for the NLR and PC were 3.0 and 400 × 10^3^/μL, respectively. Patients were scored 1 for either an elevated NLR or PC, and 0 otherwise. The NPS was calculated by summing the scores, ranging from 0 to 2. The primary endpoint was overall survival (OS) by using Kaplan–Meier analysis. Multivariate Cox regression analysis was used to identify the independent prognostic factors for OS.

**Results:**

In total, 256 metastatic UC patients were enrolled. Univariate analysis revealed that patients with either a high NLR or PC had a significantly shorter survival rate compared with those with a low NLR (*P* = .001) or PC (*P* < .0001). The median OS in patients with NPS 0, 1, and 2 was 19.0, 12.8, and 9.3 months, respectively (*P* < .0001). Multivariate analysis revealed that NPS, along with the histologic variant, liver metastasis, age, and white cell count, was an independent factor facilitating OS prediction (hazard ratio 1.64, 95% confidence interval 1.20–2.24, *P* = .002).

**Conclusion:**

The NLR and PC are independent prognostic factors for OS in patients with metastatic UC. The NPS model has excellent discriminant ability for OS.

## Introduction

Metastatic urothelial carcinoma (UC) is a lethal disease, and no major improvement has been achieved in the past 2 decades [1]. Cisplatin-based combination chemotherapy remains the standard treatment of choice. Although UC responds effectively to the cisplatin-based chemotherapy initially, most patients experience disease progression later, and mortality is inevitable. The median overall survival (OS) on cisplatin-based chemotherapy is 12–14 months [2]. Specifically, a small proportion of UC patients had a markedly effective response to chemotherapy. In previous clinical trials with durable follow-up, the 5-year survival rate of metastatic UC patients on cisplatin-based chemotherapy was approximately 10%–20% [3]. The diversity of treatment outcomes was attributed to the biologic heterogeneity of the tumor, as well as the spectrum of comorbidities of the patient, performance status, age, and disease burden. Therefore, developing a prognostic model was crucial for predicting patient outcome in daily practice and for stratifying patients in clinical trials.

Several prognostic models had been developed for predicting patient outcome in UC. Bajorin et al identified 2 independent factors associated with OS: Karnofsky performance status (KPS) < 80% and the presence of visceral (liver, lung, and bone) metastasis [4]. Based on this prognostic model, the estimated median survival time for patients with UC with 0, 1, or 2 factors was 33 months, 13.4 months, and 9.3 months, respectively. Galsky et al analyzed 384 patients on 7 prospective trials for developing a prognostic nomogram for predicting patient survival with cisplatin-based chemotherapy [5]. Five independent factors were identified and weighted differently for the point-based nomogram: leukocytosis, the number of visceral metastatic sites, the site of primary tumor, ECOG performance status, and the presence of lymph node metastasis. Notably, this is the first study that recognized and validated leukocytosis as an independent factor in advanced UC, although the underlying mechanism remains uncertain.

The inflammation process affects each step in tumorigenesis, including tumor initiation, invasion, and metastasis [6, 7]. Several serum characteristics such as the white blood cell (WBC) count, C-reactive protein (CRP), albumin, platelet count (PC), and neutrophil to lymphocyte ratio (NLR) have been investigated and validated as systemic inflammatory indicators in various types of tumors, and these factors have been found to be negatively associated with cancer survival [8–11]. Recent studies have indicated the prognostic role of a high NLR (> 3.0) and reactive thrombocytosis (> 400 × 10^3^/μL) on cancer-specific survival in preoperative UC [12, 13]; however, these have not been examined in either an advanced or metastatic setting. In addition, Ishizuka et al had developed a novel inflammation-based prognostic score, which combined the platelet count and NLR, nicely predicted postoperative survival outcome in patients with gastric cancer or colorectal cancer [14, 15]. However, this prognostic system had not been studied either in patients with UC or in those treated with systemic chemotherapy.

For this study, we conducted a retrospective analysis to evaluate the prognostic role of the combination of the platelet count and NLR in patients with advanced UC.

## Patients and Methods

### Patients and data sources

We retrospectively analyzed 303 patients with unresectable and/or metastatic UC from January 1997 to December 2014 in Kaohsiung Chang Gung Memorial Hospital, Taiwan. All patients were confirmed histopathologically. The demographic characteristics at the time of metastatic diagnosis included age, sex, ECOG performance status, the site of primary tumor, histopathologic variants, baseline renal function, the number and distribution of visceral metastasis, and first-line chemotherapy regimen. Patients lacking complete blood cell (CBC) and differentials for analysis (n = 10) or those treated with neoadjuvant (n = 6) or adjuvant chemotherapy (n = 27) were excluded. In addition, patients either without definite pathologic diagnosis (n = 3), or those with secondary non-urothelial carcinoma (n = 1) were excluded. We collected all clinical data through an electronic hospital chart review. All personal information was anonymized and de-identified prior to data analysis. This study was performed in accordance with the approval of the ethical committee.

### Treatment and study endpoint

All enrolled patients underwent systemic chemotherapy for at least one cycle of treatment. The choice of chemotherapy regimen was at the discretion of the physician. The treatment response was assessed by computed tomography (CT) and/or a bone scan according to the Response Evaluation Criteria in Solid Tumors (RECIST version 1.1). The primary endpoint of the study was OS, which was defined as the duration between metastatic disease diagnosis and the date of death. Patients without disease progression or death were censored at the date of their last follow-up for survival analysis.

### Stratification of patients

We obtained pretreatment CBC and differentials within 1 week prior to first cycle of chemotherapy. To avoid concomitant bacterial or other infectious diseases that might interfere with data accuracy, we excluded patients with either a febrile condition or any infection documented in medical charts. The NLR was determined by dividing the absolute neutrophil count with the absolute lymphocyte count. In accordance with previous studies [12, 13], the cutoff values of the NLR and PC were set to 3.0 and 400 × 10^3^/μL, respectively. We designed a novel inflammation-based score, named Neutrophil to lymphocyte ratio and Platelet count Score (NPS), for stratifying the entire study cohort. Patients were scored 1 for an elevated NLR (≥ 3.0) or PC > 400 × 10^3^/μL, and 0 if NLR<3.0 and PC <400 × 10^3^/μL. Patients were scored 2 if both NLR ≥ 3.0 and PC > 400 × 10^3^/μL. The NPS was calculated by adding the NLR and PC scores (0, 1, or 2).

### Statistical analysis

All statistical analyses were conducted using SPSS 21.0 software (SPSS Inc., Chicago, IL, USA) and SAS version 9.3 (SAS Institute Inc., Cary, NC, USA). The figures were generated using GraphPad Prism version 6.04 (GraphPad Software, La Jolla California, USA). The differences among the study groups were analyzed using the Pearson chi-squared (χ^2^) test or Fisher exact test. The estimated OS was calculated using the Kaplan–Meier method, and the differences between groups were assessed using the log rank test. Univariate and multivariate analyses of the prognostic factors for survival were performed using the Cox proportional hazards model. The hazard ratio (HR) with 95% confidence interval (CI) and *P* value were calculated to quantify the strength of the association between the prognostic parameters and survival. All tests were two-sided, and *P* < .05 was considered statistically significant.

## Results

In total, 256 patients diagnosed with unresectable and/or metastatic UC were enrolled from January 1997 to December 2014 at Kaohsiung Chang Gung Memorial Hospital. The median age was 64 years (interquartile range [IQR], 57–71 years), and the median follow-up time was 61.1 months (IQR, 26.8–150.2 months). Moreover, 172 (67.2%) patients were men, 146 (57.0%) had UC of the upper urinary tract, 128 (50.0%) had visceral metastasis, and 202 (78.9%) underwent first-line cisplatin-based chemotherapy. The median NLR at baseline was 3.83 (IQR, 2.30–7.37), and the median platelet count was 238 × 10^3^/μL (IQR, 185–305 × 10^3^/μL). Ninety-three patients (36.3%) were classified as NPS 0, 146 (57.0%) as NPS 1, and 17 (6.7%) as NPS 2.

Table 1 lists the distribution of the basic characteristics of patients in the 3 groups divided according to the NPS. Most of the characteristics compared revealed no significant differences among the 3 groups, except for visceral metastasis (yes vs no; *P* = .005), lung metastasis (yes vs no; *P* = .006), bone metastasis (yes vs no; *P* = .003), WBC count (≥10 vs <10 × 10^3^/μL; *P* < .0001), and level of hemoglobin (≥10 vs <10 g/dL, *P* = .006).

**Table 1:** Clinical characteristics of advanced urothelial carcinoma patients grouped by NPS.

	All (n, %)	NPS (n, %)			*P* value
		0	1	2	
N	256	93	146	17	
Age (year)					0.82
< 65	133 (52.0)	47 (50.5)	76 (52.1)	10 (58.8)	
≥ 65	123 (48.0)	46 (49.5)	70 (47.9)	7 (41.2)	
Gender					0.082
Female	84 (32.8)	36 (38.7)	46 (31.5)	2 (11.8)	
Male	172 (67.2)	57 (61.3)	100 (68.5)	15 (88.2)	
ECOG					0.116
0–1	201 (78.5)	76 (81.7)	109 (74.7)	16 (94.1)	
≥ 2	55 (21.5)	17 (18.3)	37 (25.3)	1 (5.9)	
Renal function (mL/min)					0.686
CCr ≥ 60	174 (68.0)	63 (67.7)	101 (69.2)	10 (58.8)	
CCr < 60	82 (32.0)	30 (32.3)	45 (30.8)	7 (41.2)	
Primary site					0.298
Bladder	101 (39.5)	29 (31.2)	65 (44.5)	7 (41.2)	
Upper tract	146 (57.0)	60 (64.5)	76 (52.1)	10 (58.8)	
Multifocal	9 (3.5)	4 (4.3)	5 (3.4)	0 (0)	
Histopathologic variants					0.295
No	161 (62.9)	57 (61.3)	96 (65.8)	8 (47.1)	
Yes	95 (37.1)	36 (38.7)	50 (34.2)	9 (52.9)	
Visceral metastasis					0.005
No	128 (50.0)	59 (63.4)	62 (42.5)	7 (41.2)	
Yes	128 (50.0)	34 (36.6)	84 (57.5)	10 (58.8)	
Liver metastasis					0.11
No	215 (84.0)	84 (90.3)	117 (80.1)	14 (82.4)	
Yes	41 (16.0)	9 (9.7)	29 (19.9)	3 (17.6)	
Lung metastasis					0.006
No	198 (77.3)	80 (86.0)	109 (77.8)	9 (52.9)	
Yes	58 (22.7)	13 (14.0)	37 (22.2)	8 (47.1)	
Bone metastasis					0.003
No	207 (80.9)	85 (91.4)	111 (76.0)	11 (64.7)	
Yes	49 (19.1)	8 (8.6)	35 (24.0)	6 (35.3)	
WBC (× 10^3^/μL)					< 0.0001
< 10	188 (73.4)	88 (94.6)	94 (64.4)	6 (35.3)	
≥ 10	68 (26.6)	5 (5.4)	52 (35.6)	11 (64.7)	
Hemoglobin (g/dL)					0.006
≥ 10	202 (78.9)	83 (89.2)	108 (74.0)	11 (64.7)	
< 10	54 (21.1)	10 (10.8)	38 (26.0)	6 (35.3)	
First-line chemotherapy					0.576
Cisplatin-based	202 (78.9)	77 (82.8)	112 (76.7)	13 (76.5)	
Carboplatin-based	44 (17.2)	13 (14.0)	27 (18.5)	4 (23.5)	
Other	10 (3.9)	3 (3.2)	7 (4.8)	0	

Abbreviation: CCr, clearance of creatinine; ECOG, Eastern Cooperative Oncology Group; NPS, neutrophil to lymphocyte and platelet count score; WBC, white blood cell count.

**Fig 1:**
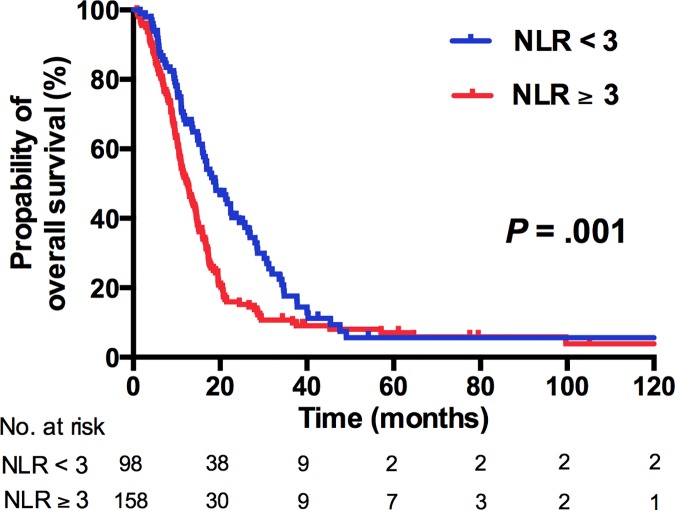
Kaplan–Meier analysis for OS according to NLR ≥ 3 or NLR < 3.

**Fig 2:**
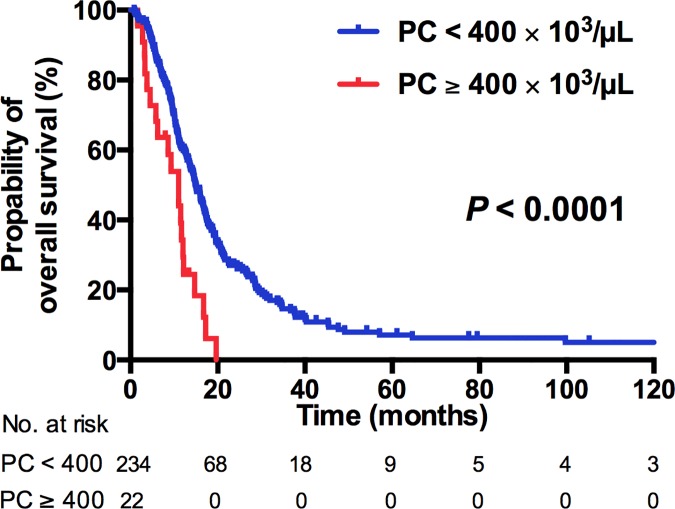
Kaplan–Meier analysis for OS according to PC ≥ 400 × 10^3^/μL or < 400 × 10^3^/μL.

**Fig 3:**
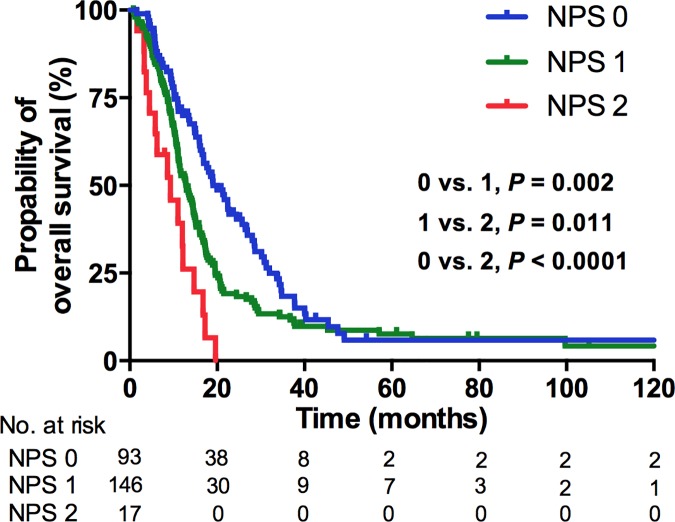
Kaplan–Meier analysis for OS according to NPS.

At the time of analysis, 214 of 256 patients (83.6%) died. The median OS was 14.5 months (IQR, 8.9–22.7 months). The median OS was 12.5 months in patients with NLR≥3, and 19.0 months in those with NLR<3 (*P* = .001, Fig 1), whereas the median OS in PC≥400 and < 400 × 10^3^/μL was 11.0 months and 15.0 months, respectively (*P* < .0001. Fig 2). Combining the NLR and PC as NPS, a high NPS demonstrated a significantly worse OS (HR 1.0, 1.59, and 3.34 for NPS 0, 1, and 2, respectively; *P* < .0001; Fig 3). The other predicting factors for OS in univariate analysis were age, ECOG performance status, the presence of histologic variants, visceral metastasis, liver metastasis, bone metastasis, WBC count, and the level of hemoglobin (Table 2).

**Table 2:** Univariate analysis of clinical parameters for overall survival.

	N	Median OS (month)	HR (95% CI)	*P*
Age (year)				0.008
< 65	133	16.8	Reference	
≥ 65	123	12.0	1.44 (1.10–1.89)	
Gender				0.076
Female	84	16.5	Reference	
Male	172	12.8	1.30 (0.97–1.73)	
ECOG				0.001
0–1	201	15.9	Reference	
≥ 2	55	10.0	1.72 (1.25–2.36)	
Renal function (mL/min)				0.953
CCr ≥ 60	174	14.6	Reference	
CCr < 60	82	13.0	0.99 (0.74–1.33)	
Primary site				0.434
Bladder	101	14.8	Reference	
Upper tract	146	13.8	0.90 (0.68–1.18)	
Histopathologic variants				0.009
No	161	15.9	Reference	
Yes	95	11.0	1.45 (1.10–1.92)	
Visceral metastasis				0.004
No	128	16.5	Reference	
Yes	128	12.1	1.48 (1.13–1.94)	
Liver metastasis				0.002
No	215	15.1	Reference	
Yes	41	11.4	1.75 (1.23–2.51)	
Lung metastasis				0.148
No	198	15.0	Reference	
Yes	58	12.0	1.26 (0.92–1.73)	
Bone metastasis				0.011
No	207	15.0	Reference	
Yes	49	11.5	1.53 (1.10–2.14)	
WBC (×10^3^/μL)				0.001
< 10	188	16.0	Reference	
≥ 10	68	9.3	1.68 (1.25–2.26)	
NLR				0.001
< 3	98	19.0	Reference	
≥ 3	158	12.5	1.60 (1.21–2.13)	
Hemoglobin (g/dL)				0.035
≥ 10	202	15.1	Reference	
< 10	54	9.6	1.41 (1.02–1.95)	
Platelet count (× 10^3^/μL)				< 0.0001
< 400	234	15.0	Reference	
≥ 400	22	11.0	2.42 (1.51–3.88)	
First-line chemotherapy				0.722
Cisplatin-based	202	14.8	Reference	
Carboplatin-based	44	11.0	0.94 (0.65–1.35)	
Others	10			
NPS				< 0.0001
0	93	19.0	Reference	
1	146	12.8	1.59 (1.18–2.13)	
2	17	9.3	3.34 (1.92–5.82)	

Abbreviation: CCr, clearance of creatinine; ECOG, Eastern Cooperative Oncology Group; NPS, neutrophil to lymphocyte and platelet count score; NLR, neutrophil to lymphocyte ratio; WBC, white blood cell count.

We selected 7 factors (age, performance status, histologic variants, liver metastasis, WBC count, hemoglobin, and NPS) for multivariate analysis by using the Cox regression model. As listed in Table 3, the best predictive factor for OS was NPS (HR 1.64, 95% CI 1.20–2.24, *P* = .002). The other significant predictors for OS were liver metastasis (HR 1.58, 95% CI 1.09–2.28, *P* = .016), poor performance status (HR 1.53, 95% CI 1.06–2.20, *P* = .023), age≥65 years (HR 1.38, 95% CI 1.02–1.88, *P* = .04), and WBC≥10 000 (HR 1.40, 95% CI 1.01–1.95, *P* = .046).

**Table 3:** Multivariable analysis of clinical parameters in relation to overall survival.

	HR	95% CI	*P*
Age (year)			
≥ 65 vs. < 65	1.38	1.02–1.88	0.04
ECOG			
≥ 2 vs. 0–1	1.53	1.06–2.20	0.023
Histopathologic variants			
Yes vs. No	1.49	1.11–1.99	0.008
Liver metastasis			
Yes vs. No	1.58	1.09–2.28	0.016
WBC (× 10^3^/μL)			
≥ 10 vs. < 10	1.40	1.01–1.95	0.046
Hemoglobin (g/dL)			
< 10 vs. ≥ 10	1.00	0.70–1.44	0.996
NPS			
1–2 vs. 0	1.64	1.20–2.24	0.002

Abbreviation: CI, confidence interval; HR, hazard ratio; ECOG, Eastern Cooperative Oncology Group; WBC, white blood cell count; NPS, neutrophil to lymphocyte and platelet count score.

## Discussion

Cancer-associated inflammation has been a well-known, poor prognostic phenomenon in various type of cancers [6, 7]. Because the NLR and elevated PC were considered as inflammation markers, a new inflammation prognostic system (NPS) had been developed and studied as a predictor of survival differences in gastric and colorectal cancer patients [14, 15]. This study is the first to validate the prognostic impact of NPS on patients with advanced UC who underwent systemic chemotherapy. Our results revealed a survival difference of 10.6 months between NPS 2 and 0, indicating that a higher NPS results in a worse prognosis. In the multivariate model, the HR of NPS remained statistically significant after adjusting for confounding factors.

In 1863, Rudolf Virchow discovered the relationship between cancer and chronic inflammation [16]. Over the past few decades, cumulative evidence has revealed that inflammation can induce tumorigenesis, including tumor initiation, malignant transformation, invasion, and distant metastasis [6]. Certain systemic inflammation markers such as CRP, WBC, albumin, NLR, and platelet count have been confirmed to be poor prognostic factors in many cancer types [5, 10, 11, 17]. Recent studies have found that a high NLR was associated with poor postoperative survival in patients with UC [18–20]. Luo et al reported that an NLR > 3 was independently associated with poor cancer-specific survival in T3 upper urinary tract urothelial cancer (UUT-UC) [19]. In a similar manner, Gondo et al retrospectively analyzed 189 patients with bladder cancer who underwent radical cystectomy, and found that a high NLR was significantly related to decreased disease-specific survival from multivariable analysis [20]. However, the majority of studies addressing the prognostic role of the NLR have been based on analyses of surgical series of bladder cancer or UUT-UC, and they had rarely focused on patients with advanced or metastatic UC. Rossi et al recently reviewed 292 patients treated with first-line chemotherapy for metastatic UC [18]. A high prechemotherapy NLR (cutoff level = 3) independently predicted poor survival outcome (HR 1.53, *P* = .01). Moreover, the dynamic change in NLR at the second or third chemotherapy cycle revealed an enhanced HR for predicting OS (HR 3.15, *P* < .0001). Our results showed that patients having a high NLR had an OS decline of 6.5 months compared with those having a low NLR. The results were consistent with the mentioned studies.

The relationship between thrombocytosis and cancer has also been explored for decades. In the previous century, Levin et al found that nearly 40% of patients with incidental thrombocytosis (with a platelet count of >400 000 per cubic millimeter) had been diagnosed with an occult cancer [21]; gastrointestinal cancer was the most common type, followed by lung, breast, and ovarian cancers. Numerous recent studies have shown that platelets actively promote tumor progression and metastasis through various mechanisms, including evading NK cells-mediated clearance, facilitating epithelial-to-mesenchymal transition, and enhancing angiogenesis [22–24]. Moreover, paraneoplastic thrombocytosis has been crucial in predicting OS in lung, colon, stomach, and ovarian cancers [9, 14, 15, 25]. Moschini et al recently found that preoperative thrombocytosis was independently associated with a poor cancer-specific survival and OS in patients with bladder cancer receiving radical cystectomy [26]. The present study is the first to investigate the prognostic role of thrombocytosis on patients with advanced UC receiving systemic chemotherapy. We discovered that patients with thrombocytosis had an increased risk of death by 242% compared with those without thrombocytosis (*P* < .0001). Therefore, paraneoplastic thrombocytosis should be considered an independent factor of the predictive model for metastatic UC.

Over the past few decades, Bajorin’s model has remained the most popular and useful prognostic model for patients with metastatic UC receiving chemotherapy [4]. Thereafter, several investigators have exerted considerable effort for exploring new prognostic factor and for revising the model. The importance of serum inflammatory markers and histopathological variants has been observed, which have been integrated into a predictive model [5, 27]. Galsky et al initially reported that leukocytosis, a systemic inflammation marker, was 1 of 5 independent factors in his predictive nomogram for metastatic UC [5]. In addition, certain authors have combined serum inflammatory markers as an inflammation-based prognostic score for predicting the survival of patients with cancer. The first score was the Glasgow prognostic score (GPS), which comprised serum CRP and albumin [17]. The predictive role of the GPS has been effectively established in several solid tumors, including lung, breast, and gastrointestinal cancers [28–30]. Ishizuka et al have recently found that simple hemogram data could predict survival precisely in patients with gastric or colorectal cancer [14, 15]. The author selected 2 parameters, the NLR and platelet count, and named the prognostic model as COP-NLR. From Kaplan–Meier analysis, patients with COP–NLR 0, 1, and 2 displayed a separate survival curve with statistical significance in gastric (*P* < .001) and colorectal (*P* < .001) cohorts. Our study is the first report to reveal the significance of the NPS on OS for patients with metastatic UC. We predicted an 11.7 months survival difference between patients with NPS 0 and 2 by using a common and simple blood test. Specifically, the platelet count cutoff value (400 × 10^3^/μL) in our study varied from that in Ishizuka’s model (300 × 10^3^/μL), which used receiver operating characteristic (ROC) curve analysis to determine the ideal cutoff level; however, we selected the platelet count cutoff level > 400 × 10^3^/μL according to the results obtained by Gakis [5]. Although a minor difference existed, we observed a similar trend and powerful results in both studies.

Our study has several limitations. First, the retrospective and nonrandomized study design limits the generalizability of the results, the evaluation of the treatment response, and a detailed analysis of adverse effects. In addition, we adjusted the intensity of chemotherapy individually based on the performance status, age, and comorbidities of the patients. Consequently, our experience reflects real-world practice, which is relevant to a similar population. Second, the NLR and PC might be influenced by several external factors, such as infection, medication, and lifestyle habits. The utility of a blood test for predicting survival should be assessed cautiously. Third, our data were retrieved from a single tertiary medical center, and were not validated externally. Analyses of the multicentric data or prospective studies are required to confirm our conclusions.

## Conclusion

In summary, our study revealed that a high NLR and platelet count were the independent prognostic factors of OS in patients with metastatic UC undergoing chemotherapy. Moreover, the new inflammation-based prognostic score, NPS, demonstrated excellent discriminant validity of OS. We recommend that the NPS be integrated into a predictive model of survival. Further confirmatory studies are necessary to validate our results.

## Supporting Information

S1 Dataset Characteristics and outcomes of the study cohort. (XLSX)
